# Regulation of arbuscular mycorrhiza development by environmental stimuli: Many roads lead to strigolactones

**DOI:** 10.1371/journal.ppat.1013555

**Published:** 2025-10-08

**Authors:** Kees Buhrman, Caroline Gutjahr

**Affiliations:** Max Planck Institute of Molecular Plant Physiology, Potsdam, Germany; University of Tübingen: Eberhard Karls Universitat Tubingen, GERMANY

The majority of land plant species recruit fungi of the phylum Glomeromycotina to form one of the most widespread plant symbioses called arbuscular mycorrhiza (AM) for mutual nutritional benefit [1–3]. Arbuscular mycorrhiza fungi (AMF) take up mineral nutrients from the soil through an extended hyphal network that forms outside the root and transports the nutrients directly into the root cortex, where they are released to the plant from highly-branched fungal structures, the arbuscules [4]. In turn, the fungi obtain photosynthetically fixed organic carbon from their host plants in the form of sugars and lipids [5,6]. AM is often described as a strategy for plants to obtain inorganic phosphate, but it also improves uptake of other inorganic nutrients, such as nitrogen, potassium, and sulfur [7–9].

The colonization of roots by AMF is largely controlled by the plant and is adjusted to the plant’s physiological state resulting from environmental conditions [10]. Under controlled experimental conditions it has, for example, been shown that root colonization is regulated by the level of phosphate, (with low levels of phosphate promoting and high levels suppressing root colonization [11,12]), as well as the level of nitrogen and other nutrients [13,14], suggesting that Liebig’s law of the minimum applies to the regulation of AM formation [15,16]. Colonization is also affected by the light environment with shade leading to a reduction of colonization via the phytochrome receptor system [17–19] and potentially also via a reduced carbon status of the plant [17,18,20].

In addition, ethylene, a plant hormone that accumulates during a number of abiotic stresses, such as anoxia, suppresses AM formation [21,22]. The influence of environmental conditions of AM development has been observed for several decades, and the molecular mechanisms regulating AM in response to environmental conditions are now beginning to be revealed.

In order to recruit AMF, many plants from liverworts to eudicots synthesize and exude carotenoid-derived compounds, known as strigolactones, into the rhizosphere [23,24]. In plant evolution, the rhizosphere function precedes its role as a plant hormone. The bryophyte *Marchantia paleacea* synthesizes and exudes the SL bryosymbiol into the rhizosphere, but does not sense strigolactones internally through the canonical strigolactone signaling pathway, as its genome does not contain a copy of the strigolactone receptor *DWARF14* (*D14*) that seems to have appeared first in the gymnosperms [25,26]*.* While this does not rule out the possibility of strigolactone perception in a non-canonical manner, as has been shown in yeast [27], developmental phenotypes of Bryosymbiol-deficient *ccd8* mutants were not described [25]. Although strigolactones comprise a collection of related compounds, of which some later biosynthetic steps are still unknown, the early catalytic steps in the biosynthetic pathway leading to active strigolactones have been well described [28]. The genes encoding the enzymes involved in these steps are *DWARF27* (*D27*), encoding a beta-carotene isomerase, *CCD7* and *CCD8* encoding two carotenoid cleavage dioxygenases, and one or multiple copies of *MORE AXILLARY GROWTH1* (*MAX1*), encoding cytochrome P450 CYP711 enzymes [28]. Mutants in *D27*, *CCD7*, and *CCD8* show reduced or delayed AM colonization in angiosperms [29–31] and an apparent absence of colonization in *M. paleacea* [25].

Nutrient conditions and other environmental factors described above lead to an alteration of strigolactone biosynthesis (see, for example, [32]). Distinct transcription factors (TFs) regulate the transcriptional response of plants to nutrient deficiencies and promote the level of strigolactone exudation as well as root colonization by AMF. The MYB-type TF PHOSPHATE STARVATION RESPONSE2 (PHR2), in rice, was found to be required for the promotion of root colonization at low phosphate conditions and to directly target the promoter of strigolactone biosynthesis genes [11,33]. PHR2 has also been associated with the nitrogen starvation response, acting in concert with NIN-LIKE PROTEIN3*,* in rice [34]. Additionally, under low phosphate conditions, the two GRAS type TFs NODULATION SIGNALING PATHWAY 1 and 2 (NSP1 and 2) dimerize and induce strigolactone biosynthesis genes [35–37]. Depending on the species, mutating *NSP1* or *2* results in a moderate to strong decrease in AM formation, and when overexpressed, *NSP2* can override high phosphate suppression of AM symbiosis in barley and *Medicago truncatula* [35,38]. NSP1 in *M. truncatula* and NSP2 in rice are proposed to bind DNA allowing the complex to activate transcription [36,37]. However, further confirmations would be helpful, as most GRAS TFs do not bind DNA. Key to promoting AM symbiosis under sufficient light conditions is the shoot-to-root mobile TF ELONGATED HYPOCOTYL5 (HY5), which is activated via the phytochrome B (PhyB) photoreceptor upon perception of red light [19,20]*.* Tomato *phyb* and *hy5* mutants display reduced root colonization by AM fungi [11,19,34,38]. Strikingly, in addition to PHR2 and NSP1, also HY5 has been shown to activate transcription of strigolactone biosynthesis genes and can directly bind their promoters [11,19,34,36,38].

In contrast, expression of strigolactone biosynthesis genes, and AM symbiosis are repressed by SUPRESSOR OF MAX2 1 (SMAX1)*,* the canonical proteolytic target of karrikin signaling [39,40]. SMAX1 is targeted by the α/β-hydrolase receptor KARRIKIN INSENSITIVE 2 (KAI2) upon perception of karrikins and unknown plant endogenous KAI2-ligands (KLs). It is then ubiquitylated by the SCF-complex, including the E3 ubiquitin ligase F-box protein MORE AXILLARY GROWTH2 (MAX2) followed by proteasomal degradation [41]. KL biosynthesis/KAI2 signaling seems to be connected to a number of environmental input pathways. For example, it seems to act downstream of phosphate starvation signaling as the expression of *KAI2* and the karrikin signaling marker gene *DLK2* is increased under low P_i_ conditions in a PHR-dependent manner, while genes with increased expression in *Ossmax1* mutants overlap with genes with reduced expression in *Osphr2* mutants [11,42]*.* Activation of strigolactone biosynthesis through the karrikin signaling pathway seems to be specific for AM-competent plants, as it does not occur (and therefore this connection must have been lost) in nonhost species such as *Arabidopsis thaliana* [43]. It also does not occur in liverworts, regardless of their capacity to form AM symbiosis [25], raising the question when during plant evolution KAI2-signaling was wired to strigolactone biosynthesis genes. On the contrary, the link between nutrient deficiency and induction of strigolactone biosynthesis is conserved in the liverwort *M. paleacea* and the hornwort *Anthoceros agrestis* [25]. We propose that strigolactone biosynthesis and exudation are a central convergence point of signaling pathways activated by distinct nutrient deficiencies and other abiotic stresses to modulate the extent of AM development.

**Fig 1:**
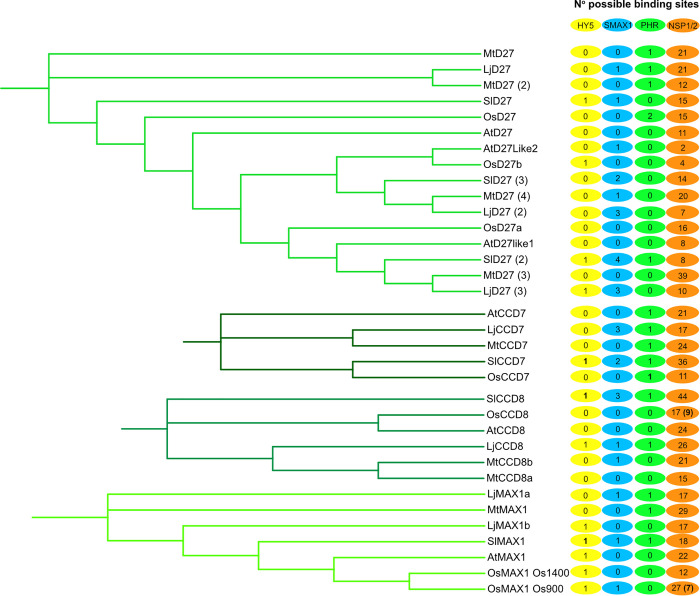
Prevalence of *cis*-elements in promoter regions of angiosperm strigolactone biosynthesis genes. Known binding sites of HY5, SMAX1, PHR, and NSP1/2 were searched in 2 kb promoter regions of *D27*, *CCD7*, *CCD8*, and *MAX1* orthologs upstream of ATG from genomes of *Solanum lycopersicon*, *Medicago truncatula*, *Lotus japonicus*, *Oryza sativa*, and *Arabidopsis thaliana*. Rice has 5 *MAX1* homologs, of which we took the promoters of *Os900* and *Os1400* to screen for transcription factor (TF) binding sites. Numbers inside TF symbols on the right of phylogenetic tree branches indicate the number of computationally identified binding sites of the respective TF in the 2 kb promoter sequence of the respective gene. Experimentally validated binding sites are depicted in bold, and for NSP1/2 in brackets [11,19,36,44]. Please note the absence of SMAX1 binding sites in promoter sequences from the genome of *A. thaliana*. It is important to note that our inability to find motifs for the different TFs in the analyzed 2 kb promoter regions does not indicate with certainty that these genes are not regulated by the respective TFs. Examples of this are *CCD7* and *CCD8* in rice, which do not exhibit possible SMAX1 binding sites in their 2 kb promoter region, but are upregulated in *Ossmax1* mutants [39]. The ‘absence’ of cis-elements in the analyzed promoter regions could be explained by i) their potential location upstream of the analyzed 2 kb region, ii) by a slightly different sequence, iii) the respective TF being promiscuous, thus binding several variations of the previously published motifs, and iv) the respective TF regulating another TF gene the product of which then binds directly to the promoter. Lastly, it is possible that in some species these TFs regulate strigolactone biosynthesis genes in concert with other proteins, without directly binding the DNA. Conversely, the binding of TFs to computationally predicted *cis*-elements needs to be validated experimentally as they could occur by chance (see, for example, the many predicted elements NSP1/NSP2 binding sites, of which many are likely false positives).

Through CHiP-seq and/or *in vivo* or *in vitro* assays in heterologous systems on wild-type and mutagenized promoter sequences, DNA-binding motifs for HY5, PHR2, NSP1, NSP2, and SMAX1 have been identified and validated in tomato (HY5, PHRs), rice (PHR2, NSP1, NSP2), *M. truncatula* (NSP1, NSP2) and *A. thaliana* (SMAX1, PHRs) [11,19, 36, 37, 44, 45, 46]. For HY5 in tomato, NSP2 and PHR2 in rice, these binding sites were experimentally validated for promoters of strigolactone biosynthesis genes [11,19]. We assessed to which extent the binding sites of these transcriptional regulators are conserved in promoter regions 2 kb upstream of ATG of *D27*, *CCD7*, *CCD8*, and *MAX1* orthologs in rice*, Lotus japonicus, M. truncatula,* tomato, and the AM nonhost *A. thaliana*. In the *CCD7* promoter sequences of all investigated species, we found the GNATATNC binding motif (P1BS) for the PHR family of TFs (Fig 1), which supports the notion that phosphate starvation-induced strigolactone biosynthesis is conserved [25]. The binding of PHR2 to the *CCD7* promoter has been validated in rice [11]. The only TF binding motif present in all analyzed sequences is that of the NSP1-NSP2 complex (Fig 1). These proteins have been shown to induce strigolactone biosynthesis in a range of angiosperms, and the presence of their DNA-binding sites in all sequences could indicate their conserved function in inducing strigolactone biosynthesis [35,38]. However, their binding site is very short (AATTT) [36,37] and promoter regions are generally AT-rich, explaining the high incidence of AATTT motives in the analyzed promoters, most of which are likely false positives. With the exception of one *DWARF27* homolog, no SMAX1 binding sites were found in promoters of strigolactone biosynthesis genes of *Arabidopsis* (Fig 1), making it tempting to speculate that strigolactone biosynthesis genes are not wired to SMAX1 in this species, although we cannot exclude that binding sites may occur in promoter sequences further upstream. Moreover, it is important to stress that even when putative SMAX1 TF binding sites are absent from promoter sequences, this does not rule out with certainty that these genes are regulated by or downstream of karrikin signaling, as in rice, the 2 kb upstream sequences of *CCD7* and *CCD8* do not contain SMAX1 TF binding sites, but are transcriptionally regulated by karrikin signaling (Fig 1, [39]).

**Fig 2:**
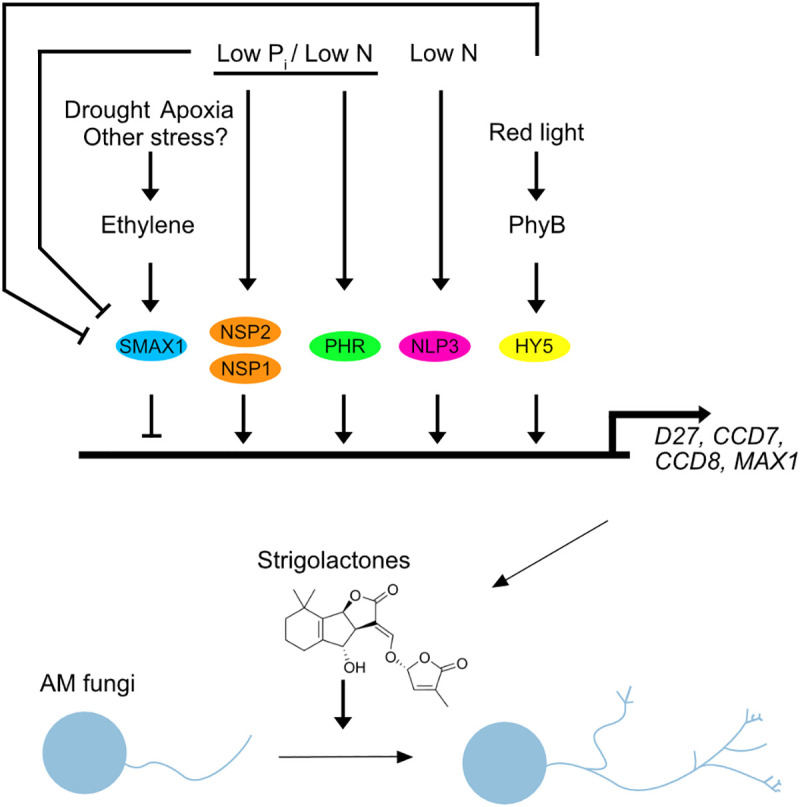
Multiple environmental conditions influence plant strigolactone biosynthesis for activation of arbuscular mycorrhiza (AM) fungi in the rhizosphere. The schematic model depicts the influence of different environmental conditions, on the activity of transcriptional regulators, which activate or repress strigolactone (SL) biosynthesis genes. PHRs, NSP1 and 2 activate SL biosynthesis genes in response to phosphate and nitrogen starvation [11,33,36], while NLP3 in concert with PHR activates SL biosynthesis genes in response to nitrogen starvation [34]. HY5 is activated by red light or at sufficient light conditions via the photoreceptor PhyB to move from the shoot to the root and induce SL biosynthesis genes [19]. By extrapolation, this explains reduced SL exudation and AM development under far-red light or shade [18]. SMAX1 suppresses the expression of strigolactone biosynthesis genes and its accumulation is promoted by ethylene and, therefore, by ethylene biosynthesis-inducing stressors [40]. Its accumulation is reduced via enhanced KAI2 signaling upon phosphate and nitrogen deficiency, potentially via the action of PHR and NSP1/2 [11,38,39,43]. The level of strigolactone biosynthesis gene expression influences the levels of strigolactone biosynthesis and exudation [11,39] and thereby hyphal branching and activity of AMF and the extent of AM formation [24].

Interestingly, ethylene negatively regulates AM symbiosis and strigolactone biosynthesis in *L. japonicus* by promoting the accumulation of SMAX1 [40]. By extrapolation, this indicates that stress conditions, which induce ethylene biosynthesis, can lead to modulation of strigolactone biosynthesis via SMAX1. A further indication of such a scenario is a report on drought stress negatively affecting strigolactone production in *L. japonicus*, independently of phosphate status [47]. Together, this positions strigolactone biosynthesis gene regulation at a convergence point of several environmental sensing pathways regulating AM symbiosis. One major gatekeeper is the transcriptional repressor SMAX1, the accumulation of which is regulated by environmental cues and nutrient status through ethylene or KL biosynthesis and/or other, yet unknown cues. For example, it was described in *Arabidopsis* that SMAX1 can also be targeted by D14 in response to osmotic stress or treatment with the synthetic strigolactone mimic GR24 [48]. In parallel, a number of TFs binding the promoters of strigolactone biosynthesis genes are activated through sensing of environmental inputs to elevate strigolactone biosynthesis (Fig 2). Although this model represents an attractive explanation of how AM may be regulated by different environmental factors it raises questions, and highlights current gaps of knowledge. For example, we do not know how several environmental sensing pathways acting in parallel are integrated to regulate strigolactone biosynthesis genes, e.g., in which situations can transcriptional repression through SMAX1 override the induction of strigolactone biosynthesis by TFs such as PHR2, and NSP1 and 2 or vice versa? Moreover, this model includes the possibility for multiple additional environmental sensing pathways and unknown TFs that link distinct nutrient deficiency stresses as well as other abiotic and potentially biotic stresses to strigolactone biosynthesis genes. Furthermore, the biosynthesis and exudation of other metabolites or the expression of other genes affecting AM symbiosis, such as the common symbiosis genes are also modulated by environmental sensing pathways and likely regulated by the same factors, as already shown for SMAX1 and PHR [11,39,40]. Mapping the molecular signals linked to nutrient deficiency stress, and understanding how they relate to other (a)biotic stresses will allow us to gain knowledge on the intricate interplay of signals that determine to which extent roots can be colonized by AM fungi.
